# An Anthropometric Study of the Facial Index Among Undergraduate Medical Students at the Institute of Medical Sciences, Banaras Hindu University, Uttar Pradesh

**DOI:** 10.7759/cureus.111443

**Published:** 2026-06-24

**Authors:** Umesh Choudhary, Md Jawed Akhtar, Mayank Gupta, Raghunath S More, Anand Mishra

**Affiliations:** 1 Department of Anatomy, Institute of Medical Sciences (IMS), Banaras Hindu University (BHU), Varanasi, IND; 2 Department of Anatomy, Indira Gandhi Institute of Medical Sciences, Patna, IND; 3 Department of Forensic Medicine, Institute of Medical Sciences (IMS), Banaras Hindu University (BHU), Varanasi, IND

**Keywords:** craniofacial anthropometry, facial index, facial phenotype, medical students, mesoprosopic, sexual dimorphism

## Abstract

Background

Assessment of facial dimensions provides valuable insights into craniofacial characteristics and population-based morphological diversity. The facial index (FI), calculated from the relationship between facial height and facial width, is commonly used to evaluate facial proportions and classify facial phenotypes. Therefore, the present study aimed to assess the FI and determine the distribution of facial phenotypes among undergraduate medical students at the Institute of Medical Sciences, Banaras Hindu University (IMS-BHU), Uttar Pradesh, India.

Materials and methods

A cross-sectional study was performed among undergraduate medical students in the Department of Anatomy at IMS-BHU, Varanasi, India. A total of 200 undergraduate medical students (104 (52.0%) males and 96 (48.0%) females) were included. Morphological facial height (nasion-gnathion distance) and bizygomatic breadth (zygion-zygion distance) were measured using a standard spreading calliper. The FI was calculated. Participants were classified into facial phenotypes according to Bannister’s classification. Data processing and statistical evaluation were carried out using GraphPad Prism. Comparisons of quantitative variables were conducted using the independent-samples t-test, whereas associations between categorical variables were assessed using the chi-square test. A p-value below 0.05 was considered indicative of statistical significance.

Results

The mean morphological facial height, bizygomatic breadth, and FI among males were 121.64 ± 6.47 mm, 138.38 ± 6.87 mm, and 88.15 ± 6.88, respectively. The corresponding values among females were 113.63 ± 4.61 mm, 132.77 ± 5.29 mm, and 85.74 ± 5.26, respectively. Male participants demonstrated significantly greater morphological facial height (p < 0.001), bizygomatic breadth (p < 0.001), and FI (p = 0.006) compared to females. The mesoprosopic phenotype was the predominant facial phenotype, observed in 69 (34.5%) participants, followed by the euriprosopic phenotype in 57 (28.5%), leptoprosopic phenotype in 30 (15.0%), and both hypereuriprosopic and hyperleptoprosopic phenotypes in 22 (11.0%) participants each. The pattern of facial phenotype distribution was comparable between sexes and did not demonstrate a statistically significant association with sex (χ² = 6.07, df = 4, p = 0.194).

Conclusion

Significant sexual dimorphism was observed in facial anthropometric measurements and the FI among undergraduate medical students at IMS-BHU, Uttar Pradesh, with the mesoprosopic phenotype being predominant in both sexes. The study provides valuable regional reference data for craniofacial assessment in clinical, forensic, and anthropological settings.

## Introduction

Anthropometry is the scientific study of human body measurements and proportions and has long served as an important tool in anatomy, anthropology, forensic science, and clinical medicine [1]. Craniofacial anthropometry, a specialised branch of anthropometry, focuses on the quantitative assessment of facial and cranial structures and provides valuable information regarding human variation, growth patterns, ethnicity, and sexual dimorphism. The human face exhibits considerable morphological diversity among populations owing to the influence of genetic, environmental, nutritional, and socioeconomic factors [2,3].

As one of the most recognisable anatomical features of the human body, the face is central to individual identity and plays a key role in social and interpersonal interactions. Accurate assessment of facial dimensions is essential in various clinical specialities, including orthodontics, maxillofacial surgery, plastic and reconstructive surgery, forensic medicine, and medical genetics [1,4]. Anthropometric evaluation of facial morphology also aids in the diagnosis of craniofacial anomalies, assessment of growth and development, treatment planning, and postoperative outcome evaluation. Furthermore, population-specific craniofacial data are invaluable in forensic identification and facial reconstruction procedures [2,5].

Among the various craniofacial parameters, the facial index (FI) is one of the most commonly used anthropometric measurements for evaluating facial morphology. It is derived by dividing the morphological facial height (nasion-gnathion distance) by the bizygomatic breadth (maximum facial width) and multiplying the result by 100 [6]. Based on this index, facial morphology is traditionally classified into five categories: hypereuriprosopic, euriprosopic, mesoprosopic, leptoprosopic, and hyperleptoprosopic. Such classification facilitates comparison of facial characteristics among different ethnic and geographic populations and contributes to the establishment of anthropometric standards [1,6].

Numerous studies have demonstrated significant variations in facial dimensions and FI among different racial and ethnic groups. Farkas LG et al., through extensive international anthropometric investigations, highlighted the existence of substantial interpopulation differences in facial morphology and emphasised the need for ethnicity-specific normative data [2]. Similarly, studies conducted in diverse populations have shown that facial dimensions vary according to genetic background, environmental influences, and geographical distribution [5,7]. These variations underscore the importance of generating regional anthropometric databases rather than relying on universal standards.

India is characterised by remarkable ethnic, cultural, and geographical diversity, resulting in considerable variation in craniofacial morphology across different populations. A recent systematic review of facial anthropometric studies in Indian populations reported significant regional differences in facial dimensions and facial phenotypes, emphasising the need for population-specific reference values for clinical and forensic applications [3].

Establishing normative facial anthropometric data is particularly important among young adults, as craniofacial growth is largely complete by this stage of life. Medical students represent a relatively healthy and homogeneous study population, with fewer confounding factors affecting craniofacial development. Despite the growing interest in facial anthropometry, there remains a paucity of data regarding FI and facial phenotype among young adults from Eastern Uttar Pradesh. Moreover, no comprehensive anthropometric study has been conducted among undergraduate medical students of the Institute of Medical Sciences, Banaras Hindu University (IMS-BHU), a premier medical institution serving a large and ethnically diverse population.

Therefore, the present study was undertaken to determine the FI and facial phenotype among undergraduate medical students at IMS-BHU, Uttar Pradesh. The findings of this study may contribute to the development of regional anthropometric standards and provide useful reference data for anatomists, forensic experts, orthodontists, maxillofacial surgeons, plastic surgeons, and researchers involved in craniofacial studies.

## Materials and methods

Study design and study setting

This cross-sectional observational study was carried out in the Department of Anatomy, IMS-BHU, Varanasi, Uttar Pradesh, India, between September 2025 and May 2026 to assess FI and facial phenotype patterns among undergraduate medical students.

Study population

A total of 200 undergraduate medical students from the IMS-BHU, Varanasi, Uttar Pradesh, were included in the study. To ensure regional representation, only students whose permanent residential address was in districts of Eastern Uttar Pradesh were eligible for inclusion. Information regarding the participants’ place of residence was obtained during data collection. The study population comprised 104 male students and 96 female students. Participants were recruited using a convenience sampling method after obtaining written informed consent and were enrolled irrespective of their year of study. All participants belonged to an age group in which craniofacial growth is considered nearly complete, thereby providing stable anthropometric measurements for analysis.

Sample size calculation

The required sample size was estimated before participant recruitment using G*Power software version 3.1.9.7 (Heinrich Heine University, Düsseldorf, Germany). The calculation was based on a comparison of facial anthropometric measurements between male and female participants using a two-tailed independent-samples t-test. Considering a medium effect size (Cohen’s d = 0.50), a significance level of 5%, and a statistical power of 80%, the minimum sample size required was 128 participants, with 64 individuals in each group. To improve the robustness of the study and compensate for potential exclusions or incomplete data, 200 participants (104 males and 96 females) were ultimately enrolled. Therefore, the final sample exceeded the calculated requirement and provided adequate statistical power for detecting sex-related differences in facial anthropometric parameters.

Ethical considerations

Ethical clearance for the study was obtained from the Institutional Ethics Committee of the IMS-BHU, Varanasi, Uttar Pradesh, India (Approval No. IMS/IEC/2025/8157). All study procedures were conducted in compliance with the principles of the Declaration of Helsinki and the recommendations of the Indian Council of Medical Research (ICMR) pertaining to research involving human participants.

Before participation, each student received detailed information regarding the purpose, methodology, and voluntary nature of the study. Written informed consent was secured from all participants prior to data collection. Participant privacy was protected throughout the study by maintaining strict confidentiality and anonymisation of all recorded information. The collected data were used exclusively for academic and research purposes, and participants retained the right to withdraw their consent at any stage without any adverse consequences.

Inclusion criteria

The study included undergraduate medical students enrolled at the IMS-BHU, who voluntarily agreed to participate and provided written informed consent. Only students whose permanent residence was located in Eastern Uttar Pradesh were included in the study. Participants were required to be apparently healthy and free from any craniofacial abnormalities that could influence facial anthropometric measurements.

Exclusion criteria

Students whose permanent residence was outside Eastern Uttar Pradesh were excluded from the study. Individuals were not considered eligible for participation if they had a previous history of craniofacial injury, congenital facial abnormalities, including cleft lip, cleft palate, craniosynostosis, or related deformities, prior orthodontic, orthognathic, facial, or maxillofacial surgical interventions, clinically evident facial asymmetry, facial pathology affecting normal morphology, or any systemic or local condition capable of influencing craniofacial measurements.

Anthropometric landmarks

Anthropometric measurements were recorded using standard anatomical landmarks commonly employed in craniofacial anthropometry.

Nasion (n)

The median point situated at the junction of the frontal and nasal bones at the root of the nose.

Gnathion (gn)

The most inferior midpoint located on the lower border of the mandible in the midsagittal plane.

Zygion (zy)

The point of maximum lateral prominence on each zygomatic arch.

These landmarks were identified by inspection and palpation before measurements were obtained.

Measurement procedure

All measurements were performed in a well-illuminated room under standardised conditions. Participants were seated comfortably on a chair with the head positioned in the anatomical position and the Frankfort horizontal plane maintained parallel to the floor. Care was taken to ensure that facial muscles remained relaxed and that the lips were gently closed during measurements.

A standard spreading calliper calibrated in millimetres was used to record facial dimensions. All measurements were obtained by the same observer to minimise interobserver variability.

Morphological facial height

Facial height was assessed as the distance extending between the nasion and gnathion landmarks. Measurements were obtained with a standard spreading calliper, ensuring accurate placement of the instrument at both reference points. All values were documented in millimetres (mm) for subsequent analysis.

Bizygomatic breadth

Maximum facial width was determined by measuring the horizontal distance between the right and left zygion landmarks, corresponding to the most prominent points of the zygomatic arches. The calliper was carefully adjusted to obtain the maximum transverse facial width without exerting excessive pressure on the soft tissues.

Calculation of FI

The FI was calculated for each participant using the following formula:

Facial index (FI) = (Morphological facial height / Bizygomatic breadth) × 100

The calculated FI was used to classify facial phenotype according to Bannister’s classification (Table 1) [8].

**Table 1:** Facial phenotype classification used in the present study according to Bannister’s classification. Note: Adapted from Prasanna LC et al. [8].

Facial index	Facial type
<79.9	Hypereuryprosopic (very broad face)
80.0-84.9	Euryprosopic (broad face)
85.0-89.9	Mesoprosopic (round face)
90.0-95.0	Leptoprosopic (long face)
>95.0	Hyperleptoprosopic (very long face)

Based on the calculated FI, each participant was categorised into one of the facial phenotypes listed in Table 1.

Statistical analysis

All collected data were entered into Microsoft Excel (Microsoft Corporation, Redmond, WA, USA) and analysed using GraphPad Prism version 10.0 (GraphPad Software Inc., San Diego, CA, USA). Continuous variables were expressed as mean ± SD, whereas categorical variables were presented as frequencies and percentages. The normality of data distribution was assessed using the Shapiro-Wilk test. Since the data were normally distributed, comparisons of morphological facial height, bizygomatic breadth, and FI between male and female participants were performed using the independent-samples Student’s t-test. The distribution of facial phenotypes between sexes was analysed using the chi-square test of independence. A two-tailed p-value of less than 0.05 was considered statistically significant. All statistical analyses were performed with a 95% confidence level.

## Results

The study population exhibited a comparable age distribution between male and female participants, with no statistically significant difference in mean age observed between the groups (p = 0.078) (Table 2).

**Table 2:** Age distribution of study participants.

Variable	Males (n = 104)	Females (n = 96)	Total (n = 200)	p-value
Age (years), mean ± SD	20.28 ± 1.11	19.90 ± 1.81	20.10 ± 1.49	0.078
Range	18.50-26.50	18.06-24.60	18.06-26.50	-

Among male participants, the mean morphological facial height was 121.64 ± 6.47 mm, with values ranging from 107.74 mm to 138.66 mm. The mean bizygomatic breadth was 138.38 ± 6.87 mm (range: 123.34-158.68 mm), while the mean FI was 88.15 ± 6.88, ranging from 75.87 to 109.98 (Table 3).

**Table 3:** Anthropometric measurements of male participants.

Parameter	Mean ± SD	Min-max
Morphological facial height (mm)	121.64 ± 6.47	107.74-138.66
Bizygomatic breadth (mm)	138.38 ± 6.87	123.34-158.68
Facial index	88.15 ± 6.88	75.87-109.98

In female participants, the mean morphological facial height was 113.63 ± 4.61 mm, with values ranging from 101.53 mm to 125.03 mm. The mean bizygomatic breadth was 132.77 ± 5.29 mm (range: 118.35-144.71 mm), and the mean FI was 85.74 ± 5.26, ranging from 73.83 to 97.88 (Table 4).

**Table 4:** Anthropometric measurements of female participants.

Parameter	Mean ± SD	Min-max
Morphological facial height (mm)	113.63 ± 4.61	101.53-125.03
Bizygomatic breadth (mm)	132.77 ± 5.29	118.35-144.71
Facial index	85.74 ± 5.26	73.83-97.88

Comparison of anthropometric parameters between sexes revealed statistically significant differences in all measured variables (Table 5). Male participants exhibited significantly greater morphological facial height than females (121.64 ± 6.47 mm vs. 113.63 ± 4.61 mm; p < 0.001). Similarly, the mean bizygomatic breadth was significantly higher among males than among females (138.38 ± 6.87 mm vs. 132.77 ± 5.29 mm; p < 0.001). The FI was also significantly greater in males (88.15 ± 6.88) than in females (85.74 ± 5.26), with the difference reaching statistical significance (p = 0.006). 

**Table 5:** Comparison of anthropometric measurements between male and female participants.

Parameter	Males (n = 104), mean ± SD	Females (n = 96), mean ± SD	p-value
Morphological facial height (mm)	121.64 ± 6.47	113.63 ± 4.61	<0.001
Bizygomatic breadth (mm)	138.38 ± 6.87	132.77 ± 5.29	<0.001
Facial index	88.15 ± 6.88	85.74 ± 5.26	0.006

The distribution of facial phenotypes among the 200 study participants is presented in Table 6. Mesoprosopic was the most prevalent facial phenotype, observed in 69 (34.5%) participants, including 34 (32.7%) males and 35 (36.5%) females. This was followed by the euryprosopic phenotype, identified in 57 (28.5%) participants, comprising 26 (25.0%) males and 31 (32.3%) females. The leptoprosopic facial type was observed in 30 (15.0%) participants, including 18 (17.3%) males and 12 (12.5%) females. Both hypereuryprosopic and hyperleptoprosopic phenotypes were observed in 22 (11.0%) participants each. The hypereuryprosopic phenotype was present in 10 (9.6%) males and 12 (12.5%) females, whereas the hyperleptoprosopic phenotype was observed in 16 (15.4%) males and 6 (6.2%) females.

**Table 6:** Distribution of facial phenotypes among study participants.

Facial phenotype	Males, n (%)	Females, n (%)	Total, n (%)	χ² value	p-value
Hypereuryprosopic	10 (9.6%)	12 (12.5%)	22 (11.0%)	6.07	0.194
Euryprosopic	26 (25.0%)	31 (32.3%)	57 (28.5%)		
Mesoprosopic	34 (32.7%)	35 (36.5%)	69 (34.5%)		
Leptoprosopic	18 (17.3%)	12 (12.5%)	30 (15.0%)		
Hyperleptoprosopic	16 (15.4%)	6 (6.3%)	22 (11.0%)		
Total	104 (100%)	96 (100%)	200 (100%)		

Chi-square analysis demonstrated that the distribution of facial phenotypes did not differ significantly according to sex (χ² = 6.07, df = 4, p = 0.194). These findings indicate that the pattern of facial morphology was broadly comparable between male and female participants.

## Discussion

The present study was undertaken to determine the FI and facial phenotypes among undergraduate medical students at IMS-BHU, Uttar Pradesh. The findings provide valuable insights into the craniofacial morphology of young adults from Eastern Uttar Pradesh and contribute to the growing body of anthropometric literature on Indian populations.

The mean morphological facial height observed in our study was 121.64 ± 6.47 mm in males and 113.63 ± 4.61 mm in females. These values are considerably higher than those reported by Pandey N et al. [9] among Nepalese medical students (109.61 mm in males and 101.34 mm in females). Our findings are more comparable to those of Jeremic D et al. [10] in the central Serbian population, who reported morphological facial heights of 121.42 ± 5.79 mm in males and 110.84 ± 5.61 mm in females. The similarity between our male participants and Serbian males is noteworthy, although Serbian females showed slightly lower values than our female participants.

The bizygomatic breadth in our study measured 138.38 ± 6.87 mm in males and 132.77 ± 5.29 mm in females. These values are higher than those reported by Shetti VR et al. [11] among Indian medical students (127.3 mm in males and 121.2 mm in females). Our findings are closer to those of Jeremic D et al. [10] in Serbia, although our values remain higher. Gupta S et al. [12] reported a facial width of 139.65 ± 7.35 mm in Haryanvi males and 134.94 ± 6.81 mm in females, which closely approximates our findings, suggesting that populations from North India may share similar facial width characteristics.

The FI in our study was 88.15 ± 6.88 in males and 85.74 ± 5.26 in females. Jeremic D et al. [10] reported FI values of 94.04 ± 7.00 in Serbian males and 92.38 ± 6.72 in females, which are higher than our findings, indicating that the Serbian population has relatively longer faces compared to the Eastern Uttar Pradesh population. FI measurements documented by Shetti VR et al. [11] in an Indian population and by Pandey N et al. [9] in Nepalese students showed close agreement with the values obtained in the current study. This similarity may reflect shared craniofacial characteristics among geographically and ethnically related South Asian populations, with a tendency toward mesoprosopic facial types.

Regarding facial phenotype distribution, our study identified mesoprosopic as the most prevalent facial type (34.5%), followed by euryprosopic (28.5%), leptoprosopic (15.0%), and both hypereuryprosopic and hyperleptoprosopic (11.0% each). Gupta S et al. [12] reported mesoprosopic as the predominant facial phenotype in the Haryanvi population (35.3% in males and 33.3% in females), which is remarkably similar to our findings. Pandey N et al. [9] also found mesoprosopic to be the dominant type (48.66% in males and 37% in females) among Nepalese medical students.

Sudikshya KC et al. [13] conducted a cross-sectional study on 156 medical undergraduates comparing Nepalese and Indian populations and reported FI values of 85.77 ± 8.31 in males and 82.97 ± 7.63 in females, which are slightly lower than our findings. In contrast to our findings, Sudikshya KC et al. [13] identified euryprosopic and hypereuryprosopic facial types as the predominant phenotypes among Nepalese and Indian participants, respectively, whereas mesoprosopic morphology was most common in the present study. This difference may be attributed to the geographic origin of the Indian participants in their study, who were from various regions rather than specifically Eastern Uttar Pradesh, further emphasising the need for region-specific normative data.

In contrast, the central Serbian population studied by Jeremic D et al. [10] demonstrated leptoprosopic as the overwhelmingly dominant phenotype (81.71%), with no hypereuryprosopic or euryprosopic types observed. This striking difference highlights the significant ethnic and geographic variation in facial morphology between European and South Asian populations. Similarly, Zhong H and Tong Q [14] reported that Tibetan youth predominantly exhibited a hypereuryprosopic facial shape (45.6% in males and 25.5% in females), which differs substantially from our findings and may reflect the distinct craniofacial characteristics of high-altitude populations.

Raveendran M [15], in a systematic review of South Asian facial anthropometry, highlighted the marked variability in craniofacial features among different populations and pointed out the limited availability of normative facial anthropometric data from many parts of South Asia, including India. This observation supports the need for regional studies aimed at establishing population-specific craniofacial reference values.

Tekin A et al. [16] studied cephalic, facial, and nasal indices in young Turkish adults and reported that males exhibited significantly greater facial width and morphological facial height than females (p < 0.001), consistent with our findings of sexual dimorphism. However, they observed that the FI did not differ significantly between sexes, and mesoprosopic types were observed exclusively in females (p = 0.005). This contrasts with our study, where mesoprosopic was the predominant type in both sexes, highlighting population-specific patterns of sexual dimorphism.

Rahimi JK et al. [17] conducted a nasofacial anthropometric study among 200 Iranian medical students and reported that the most common facial types were mesoprosopic (36%) and hyperleptoprosopic (38%), with significant sexual dimorphism observed in all nasofacial measurements (p = 0.0001). The prevalence of the mesoprosopic type (36%) in the Iranian population closely matches our finding of 34.5%, suggesting some similarity in facial proportions between Iranian and Eastern Uttar Pradesh populations despite their geographic distance. This similarity may reflect shared ancestral genetic components, environmental influences, or analogous climatic adaptations.

Bharati S et al. [18] evaluated anthropometric variability across 531 Indian populations and demonstrated substantial geographic variation in total facial index (TFI) throughout the Indian subcontinent. Their findings indicated that, although stature and nasal index effectively distinguished populations with different linguistic backgrounds, TFI showed limited discriminatory value across ethnically and linguistically diverse groups. This observation is consistent with our results, where mesoprosopic morphology predominated in both sexes despite significant differences in facial dimensions, suggesting that facial proportions may remain relatively stable within a population.

Pavlica TM et al. [19] investigated long-term changes in craniofacial morphology among adults from Vojvodina, Serbia, and reported an increase in facial height accompanied by a reduction in facial breadth over a 33-year period. These changes resulted in a progressive rise in FI, reflecting a tendency toward a more elongated facial form. Such findings are noteworthy because the present study establishes baseline anthropometric data that may facilitate future evaluation of temporal changes in facial morphology among populations from Eastern Uttar Pradesh.

Dabhi D et al. [20] investigated FI as an indicator of ethnic lineage in Upper Himalayan indigenous tribal populations from Kinnaur, Lahaul, and Spiti and reported a mean FI of 116.42 ± 9.31, with the hyperleptoprosopic facial type predominant in 93.5% of individuals. This extreme contrast with our findings, where the hyperleptoprosopic type comprised only 11.0% of participants, demonstrates remarkable ethnic and geographic variation even within the same country, underscoring the importance of population-specific normative data.

The sexual dimorphism observed in our study, with males demonstrating significantly greater facial height, bizygomatic breadth, and FI compared to females, is consistent with findings across multiple populations [9-12,16,17]. This dimorphism is likely attributable to the influence of sex hormones on craniofacial growth during puberty, with androgens promoting greater overall skeletal growth in males. However, the lack of a statistically significant association between sex and facial phenotype distribution suggests that while absolute facial dimensions differ between sexes, the proportional classification of face shape remains similar. This observation aligns with Gupta S et al. [12], who also found no significant difference in facial phenotype distribution between Haryanvi males and females, and with Sudikshya KC et al. [13], who reported that sexual dimorphism in FI was not statistically significant.

The clinical implications of these findings are substantial. In orthodontics and maxillofacial surgery, preoperative planning for orthognathic procedures requires population-specific normative data to achieve optimal aesthetic and functional outcomes. Using facial indices derived from Western populations may not always be appropriate for Indian patients, as facial proportions differ significantly across ethnic groups. The predominance of the mesoprosopic facial type in our population suggests that treatment planning for Eastern Uttar Pradesh patients should consider maintaining or achieving this proportion rather than imposing leptoprosopic or other facial types that may appear disharmonious with ethnic features.

In forensic anthropology, FI serves as an important parameter for personal identification and facial reconstruction. The establishment of population-specific normative data, as provided by this study, enhances the accuracy of facial approximation techniques and may assist in population affinity assessment of unidentified remains. Furthermore, craniofacial anthropometry is increasingly recognized as valuable in the diagnosis of congenital craniofacial syndromes, where deviations from normal facial indices may support clinical screening.

The findings of this study also have relevance for plastic and reconstructive surgery, particularly in rhinoplasty and facial contouring procedures. Understanding the normal facial proportions of the target population allows surgeons to preserve ethnic facial characteristics while addressing specific concerns. The mesoprosopic facial type, being the most common in our population, may represent a common aesthetic pattern for this region, and deviations from this pattern should be carefully evaluated.

The relatively high proportion of euryprosopic and hypereuryprosopic types in our population, totalling 39.5%, contrasts with the Serbian population, where these types were completely absent [10]. This observation supports the view that South Asian populations may have relatively broader facial configurations compared to European populations, a characteristic that may reflect genetic heritage as well as environmental and climatic influences.

Limitations of the study

Our study has a few limitations. First, we recruited a convenience sample of undergraduate medical students from a single institution. Although all participants were permanent residents of Eastern Uttar Pradesh, this specific cohort may not fully represent the region’s general population or all young adults, which limits the generalisability of our findings. Second, we relied solely on direct anthropometric measurements and did not include advanced imaging-based craniofacial assessments. To improve external validity, future multicentre studies should involve larger, randomly selected, and more diverse populations across Eastern Uttar Pradesh.

## Conclusions

Significant sexual dimorphism was observed in facial height, bizygomatic breadth, and facial index among undergraduate medical students at IMS-BHU. The mesoprosopic phenotype was the predominant facial phenotype in both sexes. These findings provide regional craniofacial reference data that may support anthropological, forensic, orthodontic, and maxillofacial applications.
